# Survey of UK clinicians’ approaches to decision making in neonatal intestinal failure

**DOI:** 10.1136/flgastro-2022-102112

**Published:** 2022-06-23

**Authors:** Pamela Cairns, Jonathan Ives, Zuzana Deans

**Affiliations:** 1 Centre for Ethics in Medicine, University of Bristol, Bristol, UK; 2 Neonatal Intensive Care Unit, University Hospitals Bristol and Weston NHS Foundation Trust, Bristol, UK

**Keywords:** INTESTINAL FAILURE, PAEDIATRIC GASTROENTEROLOGY, PARENTERAL NUTRITION, NEONATAL GUT

## Abstract

**Background:**

Outcomes for neonatal intestinal failure (IF) have improved significantly over the past two decades, however, there is no consensus for decision making among UK paediatric subspecialists.

**Objectives:**

The aim was to describe clinician’s attitudes to decision making in neonatal IF and examine variation between subspecialties.

**Methods:**

Neonatologists, paediatric surgeons and gastroenterologists were surveyed electronically. They were asked if they would recommend active or palliative care or allow the parents to decide in several scenarios; or if they considered treatment morally obligatory or impermissible.

**Results:**

Of 147 respondents, 81% of gastroenterologists would recommend active care (34.6% regardless of parental decision) for a term infant with total gut Hirschsprung’s compared with 46% and 33% of surgeons and neonatologists. No gastroenterologist would recommend palliation while 23% of both neonatologists and surgeons would. Similarly, 77% of surgeons and 73% of neonatologists would recommend palliation for a 28-week infant with IF and bilateral parenchymal haemorrhages compared with 27% of gastroenterologists.

Prognostic estimates also varied. A term baby with IF was estimated to have a survival of >80% at 5 years by 58% of gastroenterologists compared with 11.5% and 2.7% of surgeons and neonatologists. Only 11.5% of surgeons and 2.6% of neonatologist believed a 26-week preterm with IF would have a 5-year survival >60% compared with 59% of gastroenterologists.

**Conclusion:**

There is substantial variation in views about outcomes and management choices both within and between specialties; with gastroenterologists being consistently more positive. This is likely to lead to unjustified variation in counselling and parental choices.

**WHAT IS ALREADY KNOWN ON THIS TOPIC**

- The prognosis for neonatal intestinal failure has improved considerably over the past two decades. However, long-term parenteral nutrition is regarded as burdensome for children and families. Research suggests that the majority of American neonatologists and surgeons still recommend comfort care.

**WHAT THIS STUDY ADDS**

- UK neonatologists, paediatric gastroenterologists and surgeons have varying expectations of long-term survival and quality of life for infants with neonatal intestinal failure. There is inconsistency in decision making within and between different paediatric subspecialists for infants with neonatal intestinal failure. Paediatric gastroenterologists are more likely to recommend active care than neonatologist and surgeons.

**HOW THIS STUDY MIGHT AFFECT RESEARCH, PRACTICE OR POLICY**

- This survey demonstrates significant variation in how similar families may be counselled depending on the individual clinician. This justifies further research to explore the beliefs and values of clinicians, families and patients in order to develop a national ethical framework to help guide early decision making.

## Introduction

Neonatal intestinal failure (IF) (defined as a gut which is either dysfunctional or too short to absorb sufficient nutrients for survival and normal growth) is a relatively uncommon but challenging condition. It usually follows congenital abnormalities leading to a very short gut (such as gastroschisis or volvulus) or lack of function (long segment Hirschsprung’s disease) or acquired short gut secondary to necrotising enterocolitis (NEC). Active management consists of long-term parenteral nutrition (PN) combined with judicious amounts of enteral nutrition under the guidance of an intestinal rehabilitation team.^1^ This may be complicated by line infections, repeated hospitalisations, challenges of vascular access and liver disease.^2^ Parents undertake unrelenting care routines dealing with complex drug regimens, intravenous infusions and symptoms that are often nocturnal, and describe exhaustion and social isolation.^3^ Although intestinal transplantation had been hailed as a potential solution, this is currently reserved for failure of PN (due to liver failure or lack of venous access).

It had once been common to advise palliative care as the prognosis was poor and the treatment burdensome. But the prognosis has now improved dramatically with reported survival rates of more than 90%.^1 4 5^ Despite this, a survey of American clinicians reported that most would offer palliative care for neonates with severe bowel loss, with significant variation between surgeons and neonatologists.^6^

Anecdotal evidence from British parents suggests that current choices and advice is highly variable (NEC-UK, personal communication). This study aimed to describe the attitudes of UK clinicians to a variety of clinical scenarios.

## METHOD

A cross-sectional, web-based survey was developed using Survey Monkey and designed to be completed in five to ten minutes. The questions were designed to explore three areas that the literature suggests may influence clinicians, namely their belief about the clinical outcomes, the role of comorbidities and the quality of life in survivors.^6 7^

The questions were checked for face and content validity by an expert group (members of the Nutrition and Intestinal Failure Working Group (NIFWG) of the British Society of Paediatric Gastroenterology and Nutrition (BSPGHAN)). They were then piloted on a small group of clinicians and content modified to enhance clarity. The final survey is available in online supplemental file 1.

10.1136/flgastro-2022-102112.supp1 Supplementary data

The survey was fully anonymous, however, respondents had the option of giving their email address should they wish to be contacted for a subsequent qualitative study.

### Patient involvement

A parent-led charity (NEC-UK) contributed to the research question, having highlighted perceived variability. They were not involved in the design or analysis of the survey.

### Distribution

The survey was distributed to a range of relevant professional groups by the British Association of Perinatal Medicine, The British Association of Paediatric Surgery and BSPGHAN via their regular electronic newsletters The response rate was initially low. A further invitation was then emailed via one neonatologist per neonatal intensive care unit and informal networks from the surgical and gastroenterology members of the NIFWG. Ethical approval for this amendment was granted in advance.

### Analysis

Simple descriptive statistics with the data (ranked, ordinal) displayed in vertical stacked bar charts were used. A minority of those surveyed are likely to have an interest or expertise in this area. As those who responded to the survey are likely to be significantly different to non-responders, their data cannot be generalised to a wider population of clinicians. Inferential statistical analysis is therefore not meaningful.^8^

## Results

In total 147 responses were obtained, 47 from the first mailing and 100 from subsequent approach. The majority were neonatologists (75), with equal numbers of paediatric surgeons and gastroenterologists (26 of each). Twenty respondents described themselves as ‘other’—these would likely have been a combination of general paediatric trainees and specialist nurses. Comparative data for subspecialties was therefore limited to 127 respondents.

The spread of experience was similar in each subspecialty. The clinicians who participated were specialist within their fields. Seventy-four per cent of the neonatologists worked in units providing neonatal surgery, 96% of gastroenterologists worked in units providing intestinal rehabilitation services and 88% of surgeons worked in units providing bowel lengthening procedures. Thus the majority had relevant personal experience.

The first clinical question asked how likely it was that a term baby with massive gut loss secondary to a volvulus would eventually achieve enteral autonomy over a range of residual gut lengths. Although there was variation in opinion at all lengths, 20–30 cm appeared to be the point at which clinicians felt the prognosis was more positive. At less than 30 cm, 67% of respondents believed it was possible, likely or very likely for that child to achieve enteral autonomy compared with 26% believing this at less than 20 cm. Gastroenterologists were more positive than surgeons. For babies with less than 20 cm of viable bowel, 42% of gastroenterologist believed that it was unlikely or very unlikely that they would achieve enteral autonomy vs 89% of surgeons.

Four scenarios were subsequently outlined to understand the circumstances in which clinicians would counsel parents towards active or palliative care, when parental choice should be determinative and when, if ever they would be prepared to involve the courts to over-rule parental decisions (indicating that they regarded active care as either morally obligatory or impermissible).

**Figure 1:**
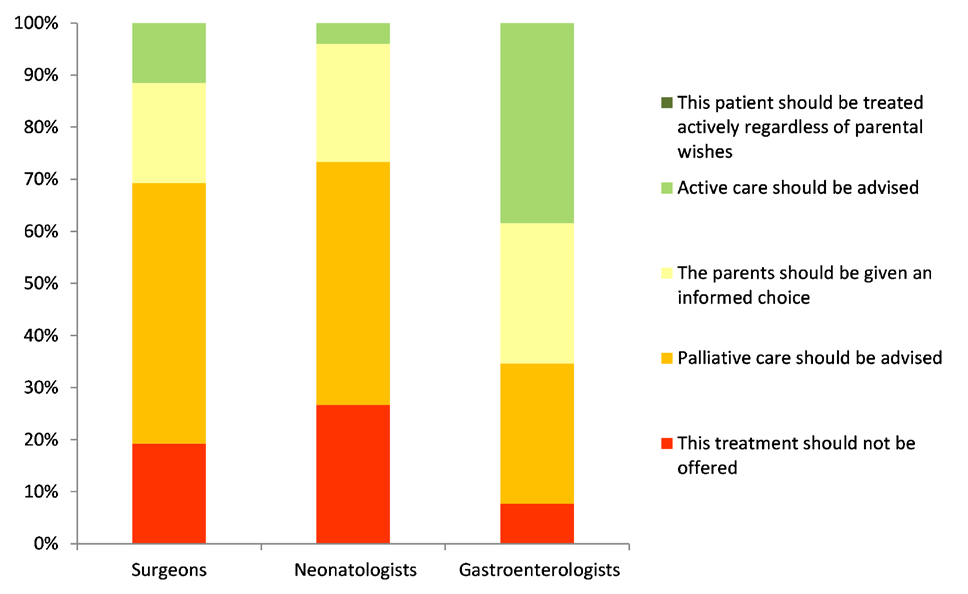
Treatment options for a 24-week gestation baby with 10 cm of small bowel remaining.

The first scenario described a 24-week gestation baby with a normal cranial ultrasound who developed severe NEC with less than 10 cm small bowel remaining after surgery. Most surgeons and neonatologists (69% and 73%, respectively) would either recommend palliative management or refuse to provide active management even if the parents request it, compared with 35% of gastroenterologists who would take the same approach (figure 1).

**Figure 2:**
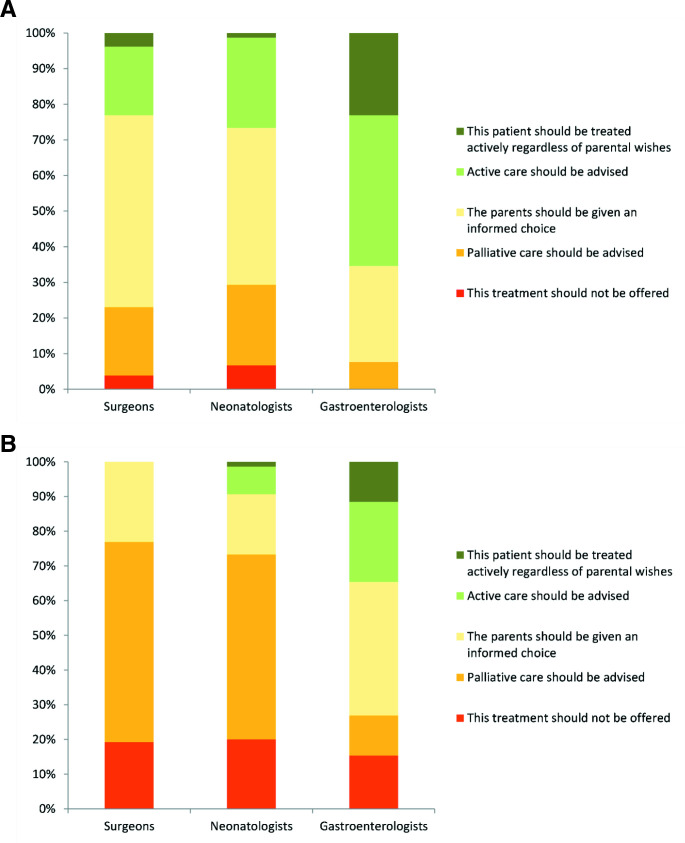
Treatment options for a 28-week gestation baby with 20 cm of remaining bowel (A) with a normal cranial ultrasound and (B) with bilateral intraparenchymal haemorrhages.

The next two scenarios described a more mature preterm baby (28 weeks) with a slightly less severe gut problem (20 cm of small bowel remaining after surgical NEC). In the first scenario he was otherwise well, in the second he had bilateral parenchymal brain haemorrhages, which are highly predictive of poor cognitive outcome and cerebral palsy.^9 10^ In the first case 42% of all respondents believed that families should be given an informed choice with 29% and 18% recommending active or palliative management respectively, although ultimately respecting parental choice. However, seven percent would overrule parental wishes to treat actively in contrast to four percent who would not make active treatment available. Although the differences between gastroenterology respondents and others remain, there is considerable variation within each subspecialty (figure 2A). In the second case, the addition of potential long-term neurodevelopmental impairment changed clinicians’ opinions about treatment options. The majority (65%) would recommend palliative management or refuse to provide active management even if the parents request it. Despite this, variation between the subspecialties remained, with 35% of gastroenterologists feeling that active care was indicated—12% being prepared to overrule a parental refusal (figure 2B).

**Figure 3:**
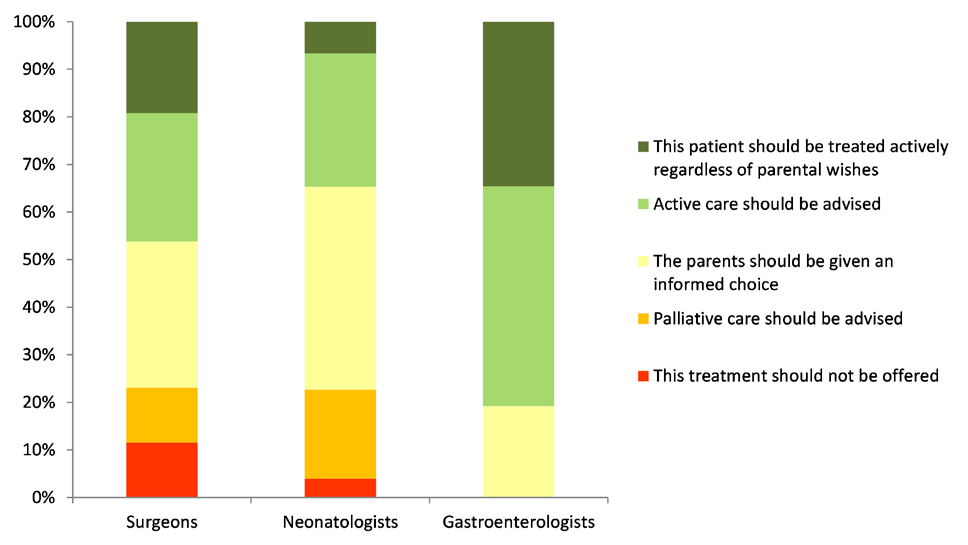
Treatment options for a baby born at 37 weeks gestation with total gut Hirschsprung’s disease.

In the final scenario, an otherwise well-term baby was diagnosed with total gut Hirschprung’s disease. Overall half (49.6%) of respondents would advise or insist on active care, 17% would either advise palliative care or not offer active treatment and one third (33.3%) would give parents an informed choice. Only 21.7% would not offer parents a choice (4% would not provide active care and 17.7% would only provide active care). In this situation, gastroenterologists would not contemplate advising palliative management and 35% felt that the baby should be treated actively regardless of parental wishes (figure 3).

Overall, the survey results indicate that respondents were more likely to recommend or insist on palliative care with increasing prematurity even in the absence of brain haemorrhages, with 17%, 22.4% and 58% advising palliation at term, 28 weeks and 24 weeks, respectively.

**Figure 4:**
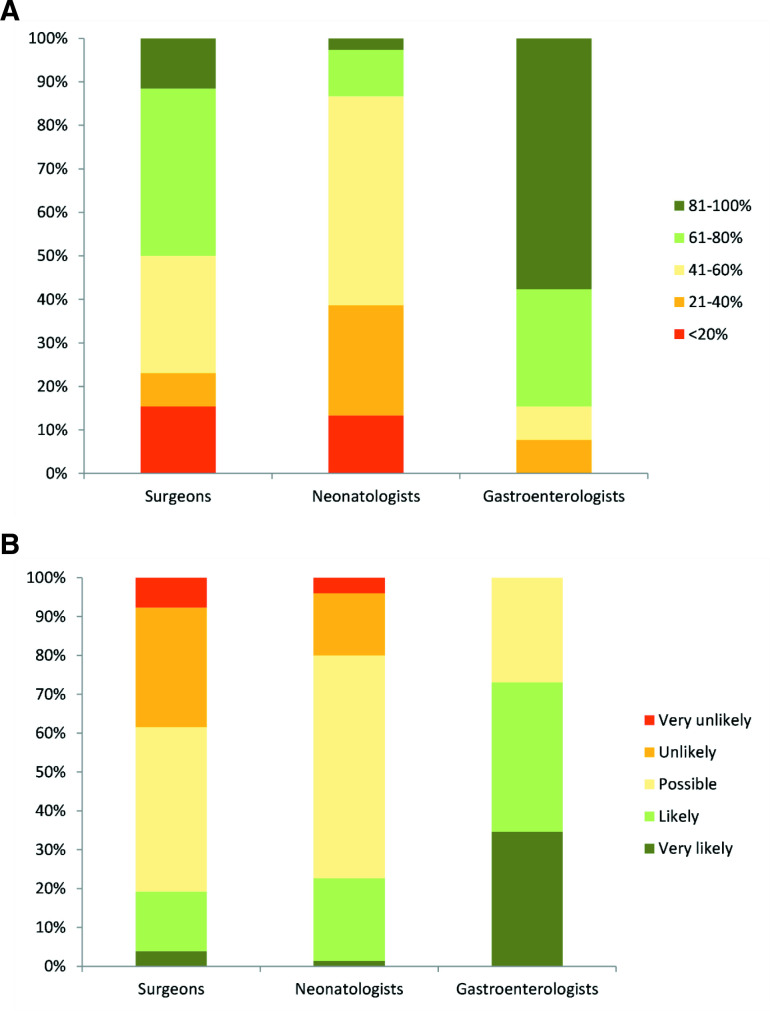
Clinicians’ estimation of (A) 5-year survival and (B) quality of life at school age of a term baby with extremely short gut secondary to in utero volvulus.

The final group of questions investigated beliefs about survival and quality of life in two scenarios. The first was that of a term baby with extremely short gut secondary to volvulus. As anticipated, gastroenterologists predicted a more positive prognosis than other professionals, with 58% estimating the child’s 5-year survival at over 80% compared with only 3% of neonatologists. In all, 86% of gastroenterologists estimated that the survival would be greater than 60% at 5 years compared with 50% of surgeons and only 13% of neonatologists (figure 4A). No gastroenterologist felt that the child was unlikely or very unlikely to have a good quality of life at school age, although 38% of surgeons and 20% of neonatologists thought that would be the case (figure 4B). There continued to be variation within specialty in addition to between specialties. For example, opinions on quality of life ranged from 4% of surgeons believing that she would be very likely to have a good quality of life and 8% believing she would be very unlikely, with a spread of opinions in between.

The same pattern of variability was observed in the case of an ex 26-week gestation baby with extremely short gut secondary to extensive NEC, although all groups believed that the outcomes would be worse in this situation. The proportion of gastroenterologists believing that that the 5-year survival was greater than 60% dropped from 85% for the term baby to 61% for the extremely preterm baby. The difference was more marked in the other specialties. While 50% of surgeons believed the 5-year survival was more than 60% in the term baby only 11% felt the same was true for the preterm compared with 13% and 3% of neonatologists. The majority of surgeons and neonatologists (62% and 78%) felt that she was unlikely or very unlikely to have a good quality of life compared with 23% of gastroenterologists.

## Discussion

The aim of this study was to describe current approaches to decision making in neonatal IF among UK clinicians. It is clear from our survey that considerable variation both between and within specialties also exists in the UK. Even in an otherwise well-term baby, for whom one would anticipate a more consistent approach, there was no consistency among clinicians regarding the appropriate direction of care. For example, families of neonates with total gut Hirschsprung’s disease may be given very different advice or treatment options depending on which individual surgeon counsels them. They may meet a surgeon who will not offer them a choice but will proceed with active care, while a similar family elsewhere will be counselled that palliative care is the only option.

Other investigators have also reported variation between subspecialties. Both Pet and Cummings reported that neonatologists were more likely to recommend comfort care and less likely to offer intestinal rehabilitation or transplantation than surgeons.^6 7^ Our study suggests that UK surgeons and neonatologists have largely similar, if heterogeneous, views while paediatric gastroenterologists are considerably more in favour of active management.

This startling variation may be partly explained by differences in understanding of the prognosis. When questioned about the 5-year survival of a term baby with IF, the difference was striking with only 3% of neonatologist believing that there was at least an 80% likelihood of the baby being alive at 5 years in contrast to 58% of gastroenterologists. Given that centres are now reporting more than 90% long-term survival, it could be that gastroenterologists are more aware of current outcomes that their surgical and neonatal colleagues.^4^ Conversely, neonatologists may be more sceptical about the long term survival data. On the other hand, the different specialties generally see these patients at different stages. The majority of neonates with IF in the UK are initially cared for on neonatal intensive care units by surgeons and neonatologists with varying input from gastroenterologists, with less than half of preterms having gastroenterology involvement in the early postoperative period.^11^ Gastroenterologists will subsequently manage the care of these patients until the end of childhood. This will result in each specialty seeing different patient groups, as gastroenterologists may not be aware of infants (particularly preterms) who do not survive their early neonatal course; while neonatologists will not see the older children who are doing well. Additionally, the centres reporting outcomes are generally specialist intestinal rehabilitation units. As Batra *et al* highlight, the true incidence and outcome is unknown due to the ‘hidden mortality’ that occurs in the neonatal period.^1^ This causes a selection bias, as neonates who did not survive to be referred would not be included. Moreover, studies have varying definitions of IF, with criteria for study inclusion and length of follow-up such that many would be considerably milder that the two cases described. So, it may be understandable that clinicians are not generally quoting 90% survival rates for severe neonatal IF. Despite these caveats, one would still judge the neonatologist views to be unduly pessimistic, as one would not anticipate such a high early death rate in term infants.

Clinicians were more likely to recommend palliative care with increasing prematurity. To some extent this is predictable, as preterm babies already have a risk of death and neuro disability that is inversely related to gestation age. This is substantially increased by severe NEC that requires surgery.^12–14^ Neonatologists are reportedly influenced by their knowledge of the adverse neurodevelopmental outcomes, while surgeons have alluded to their experience of subsequent quality of life.^6^

Although there is marked disparity between specialties in their expectations of survival and quality of life, it cannot be assumed that this completely explains the variability in attitudes. Moral uncertainty may also play a part and needs further investigation.

This study has a number of limitations. The response rate was low with 147 respondents of which 73% were consultants. The paediatric workforce survey (2015) and National Health Service hospital workforce statistics recorded a total of 647 consultants in these three subspecialties (of which 54%, 30% and 16% were neonatologists, surgeons and gastroenterologists, respectively), giving a consultant response rate of 17%.^15 16^ Low response rates to clinician surveys are common and may be indicative of workload, but limit generalisability.^17^ It is likely that only a minority of subspecialists have an interest or experience in this topic leading to a response bias. Additionally, it examined individual responses to theoretical cases. This may not be entirely reflective of real-world decision making which is more likely to involve a wider multidisciplinary team. As we did not collect occupational details of the group that described themselves as ‘other’ no inferences can be drawn about the views of general paediatricians or specialist nurses. In addition, this survey only captured the outcome of decisions, and not the reasoning behind them. However, the next phase of the study is a qualitative investigation, which will explore the beliefs and values behind the variability.

## Conclusion

There is no specific national or international guidance in this area. Despite dramatic improvements in outcomes, decision making appears highly variable and is likely to lead to unjustified disparity between treatment choices given to families. There is a need for studies reporting long term outcomes, including quality of life, that are both relevant at the point at which decisions are usually made and are significant to those involved. Further work is underway to understand the beliefs and values that underpin current decision making and the ethical values that should inform it.

## Data Availability

All data relevant to the study are included in the article or uploaded as online supplemental information.

## References

[1] Batra A , Keys SC , Johnson MJ , et al . Epidemiology, management and outcome of ultrashort bowel syndrome in infancy. Arch Dis Child Fetal Neonatal Ed 2017;102:F551–6. 10.1136/archdischild-2016-311765 28866623 PMC5739827

[2] Batra A , Beattie RM . Management of short bowel syndrome in infancy. Early Hum Dev 2013;89:899–904. 10.1016/j.earlhumdev.2013.09.001 24125822

[3] Zamvar V , Puntis JWL , Gupte G , et al . Social circumstances and medical complications in children with intestinal failure. Arch Dis Child 2014;99:336–41. 10.1136/archdischild-2013-304482 24395645

[4] Fullerton BS , Hong CR , Jaksic T . Long-Term outcomes of pediatric intestinal failure. Semin Pediatr Surg 2017;26:328–35. 10.1053/j.sempedsurg.2017.09.006 29110830

[5] Totonelli G , Tambucci R , Boscarelli A , et al . Pediatric intestinal rehabilitation and transplantation registry: initial report from a European collaborative registry. Eur J Pediatr Surg 2018;28:75-80. 10.1055/s-0037-1605349 28838002

[6] Pet GC , McAdams RM , Melzer L , et al . Attitudes surrounding the management of neonates with severe necrotizing enterocolitis. J Pediatr 2018;199:186–93. 10.1016/j.jpeds.2018.03.074 29754868 PMC6063789

[7] Cummings CL , Diefenbach KA , Mercurio MR . Counselling variation among physicians regarding intestinal transplant for short bowel syndrome. J Med Ethics 2014;40:665–70. 10.1136/medethics-2012-101269 23966424

[8] Stang A , Poole C , Kuss O . The ongoing tyranny of statistical significance testing in biomedical research. Eur J Epidemiol 2010;25:225–30. 10.1007/s10654-010-9440-x 20339903

[9] Merhar SL , Tabangin ME , Meinzen-Derr J , et al . Grade and laterality of intraventricular haemorrhage to predict 18-22 month neurodevelopmental outcomes in extremely low birthweight infants. Acta Paediatr 2012;101:414–8. 10.1111/j.1651-2227.2011.02584.x 22220735 PMC3475499

[10] Dorner RA , Burton VJ , Allen MC , et al . Preterm neuroimaging and neurodevelopmental outcome: a focus on intraventricular hemorrhage, post-hemorrhagic hydrocephalus, and associated brain injury. J Perinatol 2018;38:1431–43. 10.1038/s41372-018-0209-5 30166622 PMC6215507

[11] Jones R , Cairns P . Who looks after neonates with intestinal failure in the UK? A survey of practice. Infant 2019;15:100.

[12] Fullerton BS , Hong CR , Velazco CS , et al . Severe neurodevelopmental disability and healthcare needs among survivors of medical and surgical necrotizing enterocolitis: a prospective cohort study. J Pediatr Surg 2018;53:101–7. 10.1016/j.jpedsurg.2017.10.029 29079317

[13] Wadhawan R , Oh W , Hintz SR , et al . Neurodevelopmental outcomes of extremely low birth weight infants with spontaneous intestinal perforation or surgical necrotizing enterocolitis. J Perinatol 2014;34:64–70. 10.1038/jp.2013.128 24135709 PMC3877158

[14] Murthy K , Yanowitz TD , DiGeronimo R , et al . Short-term outcomes for preterm infants with surgical necrotizing enterocolitis. J Perinatol 2014;34:736–40. 10.1038/jp.2014.153 25144157

[15] RCPCH . Medical workforce census 2015 London: Royal College of paediatrics and child health, 2017. Available: https://www.rcpch.ac.uk/resources/workforce-census-2015-published-2017

[16] Newman TH , Parry MG , Zakeri R , et al . Gender diversity in UK surgical specialties: a national observational study. BMJ Open 2022;12:e055516. 10.1136/bmjopen-2021-055516 PMC8968535 35314455

[17] Cook DA , Wittich CM , Daniels WL , et al . Incentive and reminder strategies to improve response rate for Internet-based physician surveys: a randomized experiment. J Med Internet Res 2016;18:e244. 10.2196/jmir.6318 27637296 PMC5045523

