# Supplementary material for: Survey of UK clinicians’ approaches to decision making in neonatal intestinal failure
Source: Frontline Gastroenterol. 2022 Jun 23;14(1):13–8. doi: 10.1136/flgastro-2022-102112 (PMC9763645; doi:10.1136/flgastro-2022-102112)
Supplement: Supplementary data [file flgastro-2022-102112supp001.pdf]

# Intestinal failure test survey

**Dear colleagues,**

**Research from the US suggests that there is currently wide variation in opinion on whether to offer palliative care or active medical and surgical treatment to new-born infants with intestinal failure. In particular, it is uncertain when, if ever, the parents' views should be determinative. I wish to obtain an overview of current professional opinion in the UK.**

**I am a consultant neonatologist undertaking a part time PhD in applied Ethics. This research is being conducted as part of a Wellcome funded project with the Centre for Ethics in Medicine, University of Bristol. It involves filling in an online survey that should take around 10 minutes to complete.**

**All data will be sored and presented anonymously. Information such as your name, email address or IP address will not be routinely collected. The survey responses will be sent over a secure, SSL encrypted connection to Survey Monkey. All data will be stored in password protected electronic format on a secure server.**

**You can withdraw from the study up until the point that you submit your answers. As all of the responses are anonymous, it will not be possible to remove any individual's responses once they have completed the survey.**

**This research has been approved by the Faculty of Health Sciences Research Ethics committee, the University of Bristol.**

**If you have any questions about this study please contact pam.cairns@bristol.ac.uk.**
**Thank you for reading this.**

* 1.
If you consent to take part in the study please indicate below by checking the box

☐ I have read the information above and am happy to participate



Cairns P, *et al. Frontline Gastroenterol* 2022; 14:13–18. doi: 10.1136/flgastro-2022-102112

* 2. In your opinion how likely is it that a term baby with massive gut loss secondary to a volvulus may eventually wean off parenteral nutrition onto full enteral feeds in the following scenarios.

| | Likelihood |
|---|---|
| If the baby had less than 10cm of viable small bowel with an absent ileocaecal valve | |
| If the baby had less than 20cm viable small bowel with an absent ileocaecal valve | |
| If the baby had less than 30cm viable small bowel with an absent ileocaecal valve | |
| If the baby had less than 40cm viable small bowel with an absent ileocaecal valve | |

* 3. A four week old baby born at 24 weeks gestation develops severe necrotising enterocolitis (NEC). She is ventilated and has a normal cranial ultrasound.
At operation it is apparent that she has NEC totalis with less than 10cm of residual small bowel remaining.
In this scenario what would your opinion be of active treatment ( long term parenteral nutrition with intestinal rehabilitation +/- future gut transplant)?

- This treatment should not be offered, nor made available even if the parents request it.
- Active care should be advised but if the parents want palliative care this should be respected.
- Palliative care should be advised but if the parents want active treatment this should be respected.
- This patient should be treated actively regardless of parental wishes. If the parents refuse treatment authority should be sought from the courts.
- The parents should be given the informed choice between active or palliative treatment and the baby treated accordingly.

* 4. A four week old baby born at 28 weeks gestation develops severe necrotising enterocolitis. He was previously doing well on CPAP with no other significant co morbidities. At operation it is apparent that he has NEC totalis with less than 20cm residual small bowel remaining.
In this scenario what would your opinion be of active treatment (long term parenteral nutrition with intestinal rehabilitation +/- future gut transplant)?

- This treatment should not be offered, nor made available even if the parents request it.
- Active care should be advised but if the parents want palliative care this should be respected.
- Palliative care should be advised but if the parents want active treatment this should be respected.
- This patient should be treated actively regardless of parental wishes. If the parents refuse treatment authority should be sought from the courts.
- The parents should be given the informed choice between active or palliative treatment and the baby treated accordingly.



Cairns P, *et al. Frontline Gastroenterol* 2022; 14:13–18. doi: 10.1136/flgastro-2022-102112

* 5. A four week old baby born at 28 weeks gestation develops severe necrotizing enterocolitis. He has previously had bilateral intraparenchymal brain haemorrhages. At operation it is apparent that he has less than 20cm residual small bowel remaining.
In this scenario what would your opinion be of active treatment (long term parenteral nutrition with intestinal rehabilitation +/- future gut transplant)?

- This treatment should not be offered, nor made available even if the parents request it.
- Palliative care should be advised but if the parents want active treatment this should be respected.
- The parents should be given the choice between active or palliative treatment and the baby treated accordingly.
- Active care should be advised but if the parents want palliative care this should be respected.
- This patient should be treated actively regardless of parental wishes. If the parents refuse treatment authority should be sought from the courts.

* 6. A baby born at 37 weeks gestation is diagnosed as having total gut Hirshprungs disease. He is otherwise healthy.
In this scenario what would your opinion be of active treatment (long term parenteral nutrition with intestinal rehabilitation +/- future gut transplant)?

- This treatment should not be offered, nor made available even if the parents request it.
- Palliative care should be advised but if the parents want active treatment this should be respected.
- The parents should be given the informed choice between active or palliative treatment and the baby treated accordingly.
- Active care should be advised but if the parents want palliative care this should be respected.
- This patient should be treated actively regardless of parental wishes. If the parents refuse treatment authority should be sought from the courts.

* 7. What would you estimate the 5 year survival is of a term baby with extremely short gut secondary to in utero volvulus if she is managed on long term parenteral nutrition with intestinal rehabilitation +/- future gut transplant?

- <20%
- 21-40%
- 41-60%
- 61-80%
- 81-100%

* 8. What do you think the likelihood is for her to have a good quality of life at school age?

- Very likely
- Likely
- Possible
- Unlikely
- Very unlikely



Cairns P, *et al. Frontline Gastroenterol* 2022; 14:13–18. doi: 10.1136/flgastro-2022-102112

* 9. What would you estimate the 5 year survival is of a 26 week gestation baby with extremely short gut secondary to extensive necrotising enterocolitis if she is managed with long term parenteral nutrition with intestinal rehabilitation +/- future gut transplant?

- <20%
- 21-40%
- 41-60%
- 61-80%
- 81-100%

* 10. What do you think the likelihood for her to have a good quality of life at school age?

- Very Likely
- Likely
- Possible
- Unlikely
- Very Unlikely

Intestinal failure test survey

11. Which of the following best describes you

- Paediatric Surgeon
- Neonatologist
- Paediatric Gastroenterologist
- Other

## Intestinal failure test survey

* 12. How many years have you been in a consultant post

- I am a trainee
- <5 years
- 5-10 years
- 10-20 years
- 20 – 30 years
- >30 years
- I am retired

13. Do you  work in a unit undertaking any of the following?

| | |
|---|---|
| Neonatal Surgery | |
| Bowel Lengthening Surgery | |
| Intestinal Transplant | |

## Intestinal failure test survey

* 14. How many years have you been in a consultant post

- I am a trainee
- <5 years
- 5-10 years
- 10-20 years
- 20 – 30 years
- >30 years
- I am retired

15. Do you work in a unit with neonatal surgery?

- Yes
- No

## Intestinal failure test survey

* 16. How many years have you been in a consultant post

- I am a trainee
- <5 years
- 5-10 years
- 10-20 years
- 20 – 30 years
- >30 years
- I am retired

17. As a gastroenterologist

| | |
|---|---|
| Do you work in a transplant centre | |
| Does your unit care for children on home PN | |
| Does your unit run an intestinal rehabilitation service | |

Intestinal failure test survey

**Thank you for participating in this survey.**

**I understand that these decisions are complex and cannot be fully captured in a brief survey. The next phase of the research will involve semi-structured interviews with a range of health professionals. If you may be interested in taking part please give your email address below (this will not be linked to your answers). I can then contact you with further information when this phase is starting.**

18. Contact email address
